# Evaluation of micro-RNA in extracellular vesicles from blood of patients with prostate cancer

**DOI:** 10.1371/journal.pone.0262017

**Published:** 2021-12-31

**Authors:** Jiyoon Kim, Siwoo Cho, Yonghyun Park, Jiyoul Lee, Jaesung Park

**Affiliations:** 1 School of Interdisciplinary Bioscience and Bioengineering, Pohang University of Science and Technology, Pohang, Gyeong-buk, Republic of Korea; 2 Department of Mechanical Engineering, Pohang University of Science and Technology, Pohang, Gyeong-buk, Republic of Korea; 3 Department of Urology, Seoul St. Mary’s Hospital, College of Medicine, The Catholic University of Korea, Seoul, Republic of Korea; Children’s National Hospital, UNITED STATES

## Abstract

Extracellular vesicles (EVs) contain various types of molecules including micro-RNAs, so isolating EVs can be an effective way to analyze and diagnose diseases. A lot of micro-RNAs have been known in relation to prostate cancer (PCa), and we evaluate miR-21, miR-141, and miR-221 in EVs and compare them with prostate-specific antigen (PSA). EVs were isolated from plasma of 38 patients with prostate cancer and 8 patients with benign prostatic hyperplasia (BPH), using a method that showed the highest recovery of RNA. Isolation of EVs concentrated micro-RNAs, reducing the cycle threshold (Ct) value of RT-qPCR amplification of micro-RNA such as miR-16 by 5.12 and miR-191 by 4.65, compared to the values before EV isolation. Normalization of target micro-RNAs was done using miR-191. For miR-221, the mean expression level of patients with localized PCa was significantly higher than that of the control group, having 33.45 times higher expression than the control group (p < 0.01). Area under curve (AUC) between BPH and PCa for miR-221 was 0.98 (p < 0.0001), which was better than AUC for prostate-specific antigen (PSA) level in serum for the same patients. The levels of miR-21 and miR-141 in EVs did not show significant changes in patients with PCa compared to the control group in this study. This study suggests isolating EVs can be a helpful approach in analyzing micro-RNAs with regard to disease.

## Introduction

Micro-RNAs (miRNAs) are small noncoding RNA molecules that regulate gene expression at the posttranscriptional level [1]. They play a regulatory role by binding to 3’ UTR of transcripts and are involved in many developmental and physiological processes [2]. Abnormal expression pattern of micro-RNAs has been related to disease such as cancer, and lots of research has been conducted to apply micro-RNAs diagnostically [3].

In biological fluids such as blood, urine, and saliva [4–6], micro-RNAs are known to exist in one of the forms that involve extracellular vesicles (EVs) or proteins like high-density lipoprotein or Argonaute2 protein [7, 8]. These structures are thought to provide protection or stabilization to the miRNAs from degradation by RNases in biological fluids [9–11]. Among these structures, EVs are secreted from almost all types of cells, carrying a wide range of information which is derived from original cells in the form of proteins, mRNA, and miRNAs [12–14]. Recent studies revealed that the acidic microenvironment surrounding a tumor increases the release of exosomes and the expression of tumor-related molecules in them [15]. Since EVs reflect their cells of origin in the diseased state or healthy state, detecting and investigating the molecules in EVs from body fluids can be a good strategy for liquid biopsy.

In diagnosis of prostate cancer (PCa), the level of prostate-specific antigen (PSA) in blood gives primary information about the possible presence of prostate cancer [16], and its concentration in serum more than 4 ng/ml calls for further examination for PCa. However, because of its low specificity, PSA has frequently brought about the overdiagnosis and overtreatment, causing unnecessary biopsies for patients [17]. Therefore, there have been needs for another markers which can discriminate BPH from the early stage of prostate cancer more accurately [7].

The aim of this study is to investigate how micro-RNAs in EVs would be effective in comparison with PSA, and we test three miRNAs, miR-21, miR-141, and miR-221 by isolating EVs from plasma, and compare the result with the level of PSA regarding their diagnostic performance in patients with prostate cancer [18, 19].

## Materials and methods

### Preparation of plasma

Plasma was provided by the Korea Prostate Bank through Infrastructure Project for Basic Science of the Ministry of Science and ICT, Korea. Patients were included only if they had no previous history of prostatic malignancy and had not received androgen deprivation therapy, and they were retrieved randomly and consecutively during sampling. Patients were grouped in consideration of TNM staging as follows: a localized prostate cancer group (N0 stage), a local advanced prostate cancer group (N1 stage), and metastasized group (M1 stage). Plasma was obtained right before the surgical intervention for prostate cancer (PCa) or benign prostatic hyperplasia (BPH).

Plasma samples were obtained by phlebotomy from patients with BPH (n = 8), localized prostate cancer (n = 15), local advanced prostate cancer (n = 16), and metastasized prostate cancer (n = 7) (S1 Table). After sampling, the plasma was pre-cleaned at 800 × g force for 10 min and 2000 × g force for 20 min sequentially, then stored at -70°C until use. The plasma was thawed and filtered through a 0.8-μm syringe filter right (Advantec^®^ DISMIC-25CS) before use.

### EV isolation from plasma

Traditional ultracentrifugation was conducted by pelleting 250 μℓ of pre-cleaned plasma samples at 100,000 × g twice; the pellets were re-suspended in 40 μℓ of 1× phosphate-buffered saline (PBS) [20].

For Exoquick^™^ method (System Biosciences, Inc.), the Exoquick solution 63 μℓ was added to the pre-cleaned plasma 250 μℓ to precipitate vesicles, and the mixture was incubated for one hour at 4°C and then centrifuged 1600 × g for 30 min; the precipitated vesicles were also mixed with 40 μℓ of 1× PBS.

For Exo2D method (EXOSOME plus, Inc.), Exo2D solution 50 μℓ was mixed with pre-cleaned plasma 250 μℓ, put on a shaker for 30 minutes for complete mixing, and centrifuged at 3,000 × g for 30 min at 4°C to induce phase separation. The upper phase and the lower phase were distinguished by a clear, transparent border within the solution mixture. The upper phase, which takes up the majority of volume and sequesters proteins [21], was gently removed by micro-pipetting. Then the remaining lower phase that is composed of aggregated EVs in bottom was re-suspended in 40 μℓ of 1× PBS [21].

### Bioanalyzer

Qualitative profile of RNA was measured using an Agilent 2100 Bioanalyzer small RNA kit (Agilent Technology). To compare small RNA profile of each isolation method, RNAs in EVs were isolated from 500 μℓ of plasma by ulracentrifugation, ExoQuick, or Exo2D, re-suspended in 10 μℓ of nuclease-free water, and then loaded on the chip, respectively.

### Transmission Electron microscopy

Transmission Electron microscopy (TEM) was conducted to identify EVs from plasma. The protein concentration in EVs was prepared as 200 ng/μℓ, and 7 μℓ of each sample was placed on a formvar/carbon TEM grid (FCF300-cu, Electron Microscopy Science). Then, it was stained with 7 μℓ of 2% uranyl acetate for 10 s. The sample on the grid was air-dried completely for more than 30 min and imaged using an Erlangshen ES1000W CCD camera (Gatan inc.) at 60-kV operating voltage.

### Cell culture

Prostate cancer cell line, LnCap [22] was obtained from ATCC (Manassas, VA, USA) and cultured in RPMI1640 media (Gibco) with 10% fetal bovine serum (Hyclone) and 1% penicillin-streptomycin (Anti-Anti; Gibco).

### Western blot

EVs and LnCap cells [22] were suspended in RIPA buffer containing proteinase K inhibitor. The concentration of proteins was measured by the BCA assay; 60 μg of proteins was prepared from pre-cleaned plasma, EVs and LnCap cells for Western blot. Denatured proteins were separated using SDS polyacrylamide gel electrophoresis at 80 V for 150 min. Then, the samples were transferred to a PVDF membrane at 390 mA and 4°C for 2 hours. The PVDF membrane was treated with blocking solution, then reacted with 0.2 mg/mℓ Calnexin, CD63, CD9, CD81, and 0.1 mg/mℓ actin primary antibodies (sc11397, sc5275, sc51575, sc166029 and sc81178, Santa Cruz). Then, 0.1 mg/mℓ HRP conjugated secondary antibodies (anti-rabbit or anti-mouse IgG HRP, Santa Cruz) were applied for 1 hour, and the target protein was detected by adding chemiluminescent substrate (Amersham Pharmacia Biotech).

### Nanoparticle tracking analysis

Isolated EVs were analyzed using nanoparticle analysis (ExoCope mono^™^, software ver. 0.5, EXOSOME plus, Inc.) to measure the size and number of particles with scattered light. Recording time was 30 sec, and measurements were repeated three times. The intensity threshold was set to 20 Npixel (quadrature noise level in digital number per pixel); minimum tracked particle size was set to 50 nm; minimum particle distance was set to 5.7 pixel. Each sample was diluted with filtered 1× PBS to 1/50 ~1/100 concentration.

### RNA isolation

RNA from EVs was isolated as follows. First, each sample was added with 1mℓ Trizol^™^ (Invitrogen) and 200 μℓ chloroform, and then it was vortexed and centrifuged at 16,000 × g for 10 min. Next, the aqueous phase was removed, and the lower phase is mixed with an equal volume of isopropanol and stored at −20°C overnight. Then, the samples were centrifuged again at 16,000 × g for 10 min. The supernatant was discarded, and the pellet was washed with 75% ethanol and dissolved in nuclease-free water. RNA concentration was measured using SimpliNano (GE Healthcare).

### Reverse transcription quantitative PCR (RT-qPCR)

Synthesis and amplification of cDNA were conducted using miRNA-specific TaqMan microRNA Expression Assays (4427975, ThermoFisher^™^) for hsa-miR-103-3p (ID: 000439), hsa-miR-16-5p (ID: 000391), hsa-miR-191-5p (ID: 002299), hsa-miR-21-5p (ID: 000397), hsa-miR-141-3p (ID: 000463), and hsa-miR-221-3p (ID: 000524).

First, a total 80 ng of RNA was mixed with 3 μℓ of TaqMan MicroRNA Assay RT primers (4427975, ThermoFisher^™^), 3μℓ of 5× Reaction Buffer (ImProm II; Promega), 1.2 μℓ of 25 mM MgCl_2_ (Promega), 1 μℓ of 10 mM dNTP mix (Promega), 0.5 μℓ of ribonuclease inhibitor (RNasin; Promega), 0.5 μℓ of reverse transcriptase (ImProm II; Promega), and nuclease-free water for 15 μℓ reaction of reverse transcription. The reverse transcription reaction was performed as follows: 16°C for 30 min, 42°C for 30 min, 85°C for 30 min, cooling at 4°C.

Then, 1 μℓ of reverse transcription products was mixed with 1 μℓ of TaqMan^®^ MicroRNA Assay TM primers (4427975, ThermoFisher^™^), 10 μℓ of Taqman^®^ Universal PCR Master Mix, no AmpErase^®^ UNG (4324018, ThermoFisher^™^), and nuclease-free water for 20 μℓ reaction of amplification. The qPCR reaction was performed as follows: 95°C for 10 min, 45 repeated cycles of 95°C for 15 s, and then 60°C for 1 min.

### Determination of endogenous normalizers by NormFinder algorithm

Three putative normalizers (miR-16, miR-191 and miR-103) which were searched through literature review were tested for use in EVs from patients with prostate disease [23, 24]. Samples were recruited randomly and consecutively and then divided into a group with BPH (8 patients) and the other group with prostate cancer (9 patients) to find the most stable reference.

RT-qPCR reactions were performed for miR-16, miR-191 and miR-103 in EVs from plasma. Ct values were converted to relative quantities (RQ) [25] with RQ = 1/(2^(*A*—*B*)^), where *A* is Ct of the sample, and *B* is Ct of the sample that has the lowest Ct in the same group. RQ values were entered in an Excel spreadsheet and used as inputs for the NormFinder program [26]. The PCa and BPH group were designated as a different integer in the Excel spreadsheet. Then the NormFinder macro, Excel Add-in, was executed; this analysis yielded stability value [27], intragroup variation and intergroup variation.

### Analysis of RT-qPCR results

All RT-qPCRs were performed in triplicate. Ct values were analyzed by the 2^-(ΔΔCt)^ method [28, 29]. The level of each miRNA was normalized by the level of miR-191-5p. Samples from BPH patients were regarded as a control. Fold changes were calculated using the 2^-(ΔΔCt)^ values.

### Statistics

Data were analyzed using SPSS statistics 24 (IBM), OriginPro 2015 (Origin lab), and Prism (GraphPad). One-way analysis of variance (one-way ANOVA) was used to compare the level of each miRNA among patient groups. Student’s t-test was applied to analyzing two groups. P values < 0.05 were considered as statistically significant.

### Statement of ethics

This study protocol was approved by the Institutional Review Board at Pohang University of Science and Technology (No. PIRB-2016-E027), which abides by the Declaration of Helsinki; the study was implemented in agreement with the guidelines and principles of the Declaration.

All participants conferred informed consent for their participation into the study and allowed written publication of the results.

## Results

### Selection of EV isolation method and characterization of EVs from plasma

**Fig 1:**
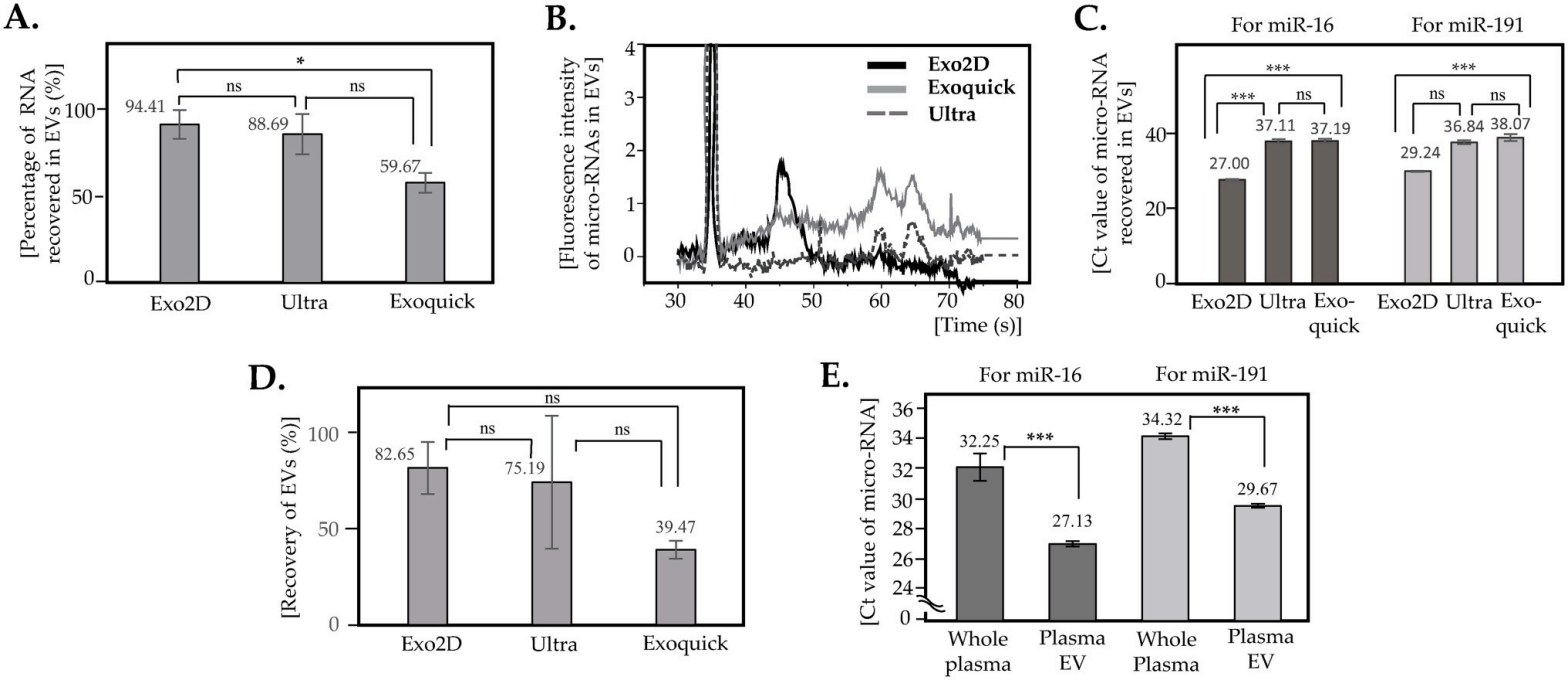
Retrieval of RNA in EVs from plasma. (A) Percentage of RNA in EVs recovered from plasma by each isolation method. The amount of RNA in EVs was divided by the amount of RNA in precleaned plasma with same volume. Error bars: ± 1 s.d., n = 3, *: p < 0.05, ns: not significant (One-way ANOVA with Tukey’s post-hoc analysis). (B) Qualitative profiles of micro-RNAs by small RNA bioanalyzer for each EV isolation method. EVs were isolated from same volume of plasma, then RNA was resuspended in the same volumes of nuclease-free water at the same condition. (C) Quantitative comparison of EV isolation techniques. EVs were isolated from 250 μℓ of plasma. RNA input = 100 ng, error bars: ± 1 s.d., n = 3, ***: p< 0.001, ns: not significant (One-way ANOVA with Tukey’s post hoc analysis). (D) Recovery of EVs isolated by each method. The number of particles in EVs was divided by the number of particles in the precleaned plasma for each isolation method. Particle numbers were measured by Nanoparticle tracking analysis. Error bars: ± 1 s.d., n = 3, ns: not significant (One-way ANOVA with Tukey’s post-hoc analysis). (E) Effect of EV isolation on micro-RNA in plasma. EVs were isolated from 250 μℓ plasma by Exo2D^™^. RNA input = 90 ng; error bars: ± 1 s.d., n = 3; ***: p < 0.001; *: p < 0.05 (Student’s t-test).

Before analyzing patient samples, we evaluated three EV isolation methods, which were traditional ultracentrifugation and two commercial kits, Exoquick^™^ and Exo2D^™^ (Fig 1A–1D). The quantity and quality of RNA in EVs were measured for each method. The amount of RNA recovered in EVs took up 94.41% of total RNA from pre-cleaned plasma for Exo2D^™^, 88.69% for ultracentrifugation, and 59.67% for Exoquick^™^ (Fig 1A). Qualitative analysis of small RNA showed that RNA from EVs isolated by Exo2D^™^ showed higher profile in the region between 40 seconds and 50 seconds than RNA from other two methods, ultracentrifugation and ExoQuick^™^ (Fig 1B). To compare the expression level of specific miRNAs, the level of expression was identified for miR-16 and miR-191 from EVs by each isolation method [30]. With the same RNA input, RNA in EVs by Exo2D showed the lowest threshold value (Ct) or the fastest amplification for both miR-16 and miR-191, suggesting the presence of proper amount of micro-RNAs (Fig 1C). When EV recovery was examined for each method, it was 82.65% for Exo2D, 75.19% for ultracentrifugation, and 39.47% for ExoQuick (Fig 1D). EV isolation made enrichment of the selected micro-RNAs with Ct value of miR-16 decreasing from 32.25 to 27.13 and miR-191 from 34.32 to 29.67, respectively (Fig 1E).

**Fig 2:**
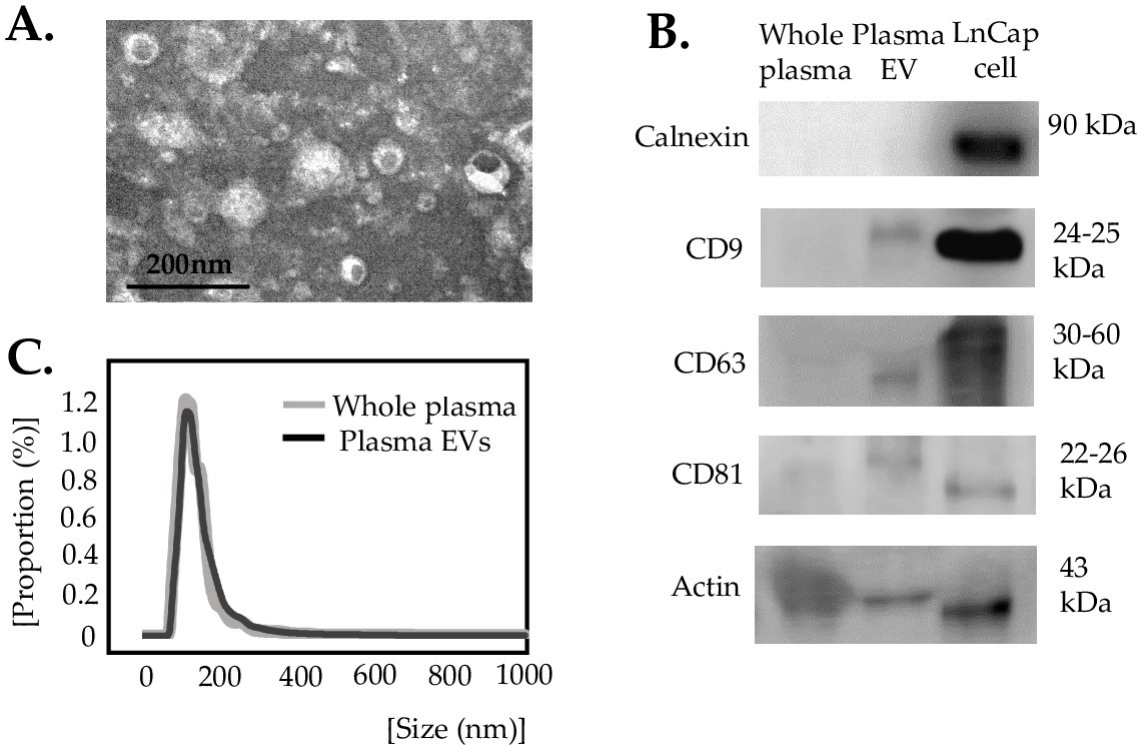
Characterization of EVs from plasma. EVs were isolated by Exo2D^™^. (A) TEM image of EVs. A scale bar with 200 nm length. (B) EV markers identified by Western blots. LnCap, a prostate cancer cell line, was used as a positive control. (C) Size distribution of EVs measured by Nanoparticle Tracking Analysis. Proportion of particles at each size in total population of particles was represented as percentage.

EVs isolated by Exo2D^™^ were characterized for their shapes, protein markers, and size (Fig 2) [31]. Circular vesicular structures that ranged from 30 nm to 400 nm in diameter were identified using transmission electron microscopy (Fig 2A). Tetraspanins such as CD9, CD63 and CD81 were identified in isolated EVs (Fig 2B). Calnexin, a negative marker for EVs, was not identified in isolated EVs [32]. Most EVs were under 400 nm in diameter when measured by Nanoparticle Tracking Analysis (Fig 2C) (ExoCope mono^™^, EXOSOME plus, Inc.).

### Finding normalizer for miRNA in EVs from plasma

**Fig 3:**
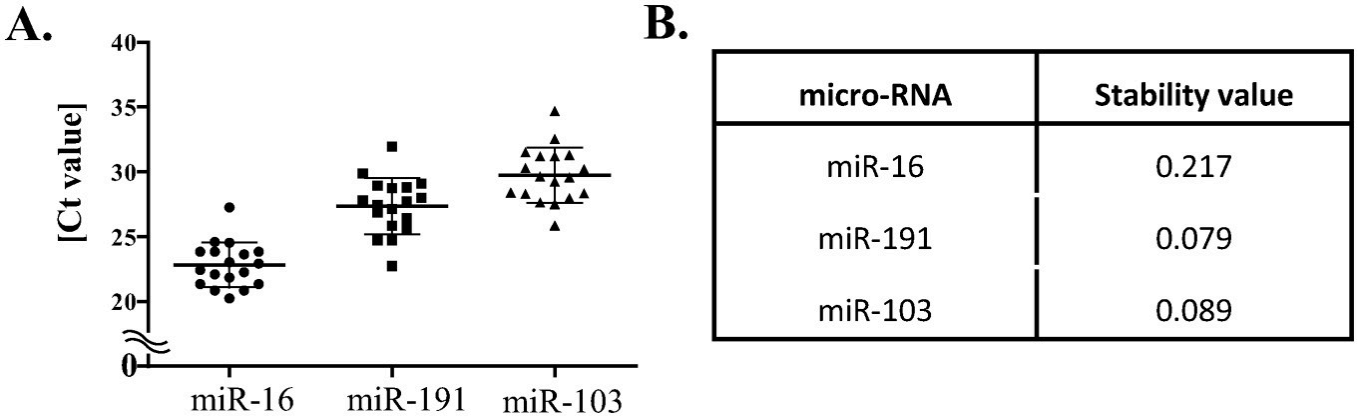
Selection of reference miRNA in EVs from plasma of patients with prostate disease. (A) RT qPCR results of miR-16, miR-191 and miR-103. Each dot represents a mean Ct value of one sample. A middle lines shows a mean value for each micro-RNA, and upper and lower lines show standard deviations. (B) Stability value of three micro-RNAs by Normfinder.

In isolated EVs, miR-16, miR-191 and miR-103 were tested for their adequacy as endogenous references in EVs from plasma of patients with prostate disease (Fig 3) [30]. Normfinder program [25, 27] was used to analyze the RT-qPCR results of three putative references. Mean Ct values were 22.82 for miR-16, 27.36 for miR-191, and 29.74 for miR-103. Among them, miR-191 had the lowest stability value, which means it had the best stability among reference candidates in this experimental condition.

### Evaluation of miR-21, miR-141, and miR-221 in EVs from plasma and comparison to PSA level

**Fig 4:**
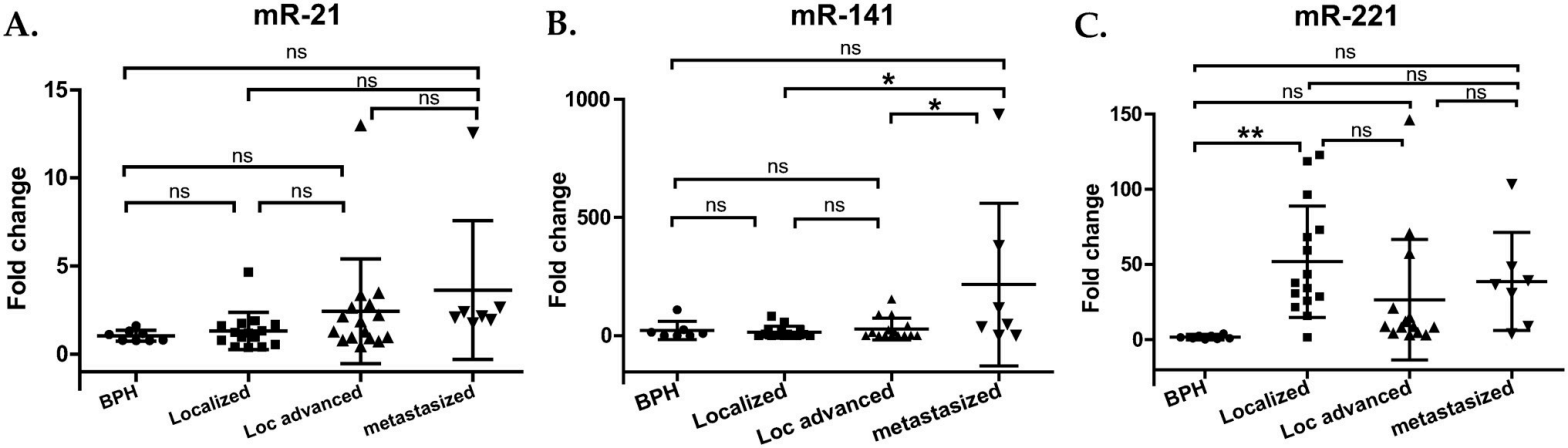
Analysis of normalized miR-21, miR-141, and miR-221 in plasma of patients with prostate cancer (PCa). The level of each micro-RNA was normalized by the level of miR-191. Each dot represents 2^-(ΔΔCt)^ or fold change for each sample; ΔΔCt = ΔCt of a sample—mean ΔCt of a control group, ΔCt = mean Ct of target gene—mean Ct of reference gene. Middle lines: means; upper and lower lines: ± 1 standard deviation. “Localized” denotes patients with localized PCa, “Loc advanced” means patients with local advanced PCa, and “metastasized” signifies patients with metastasized PCa. (A) Fold change of miR-21 in plasma EVs. ns: not significant (B) Fold change of miR-141 in plasma EVs. *: p < 0.05 (C) Fold change of miR-221 in plasma EVs; **: p < 0.01 (One-way ANOVA with Tukey’s post hoc analysis).

The levels of miR-21, miR-141, and miR-221 were analyzed in EVs from plasma of patients with prostatic cancer (Fig 4). Patients with benign prostatic hypertrophy (BPH) were used as a control group to evaluate the patients with prostatic cancer. For miR-21, patient groups with PCa did not show a meaningful change in comparison with the control group (Fig 4A). For miR-141, any group with PCa did not give a statistically significant change compared to the control group (Fig 4B). However, the group with metastasized PCa had 7.60 times higher expression than the local advanced PCa group (p < 0.05) and 13.58 times higher expression than the localized PCa group (p < 0.05) (Fig 4B). For miR-221, patient group with localized PCa showed 33.45 times higher expression than the control group (p < 0.01), providing distinctive information for less progressed state (Fig 4C). The group with local advanced PCa had 17.08 times higher expression of miR-221 than the control group, and the metastasized group showed 24.92 times higher expression than the control group, but these changes were not statistically significant (Fig 4C).

**Fig 5:**
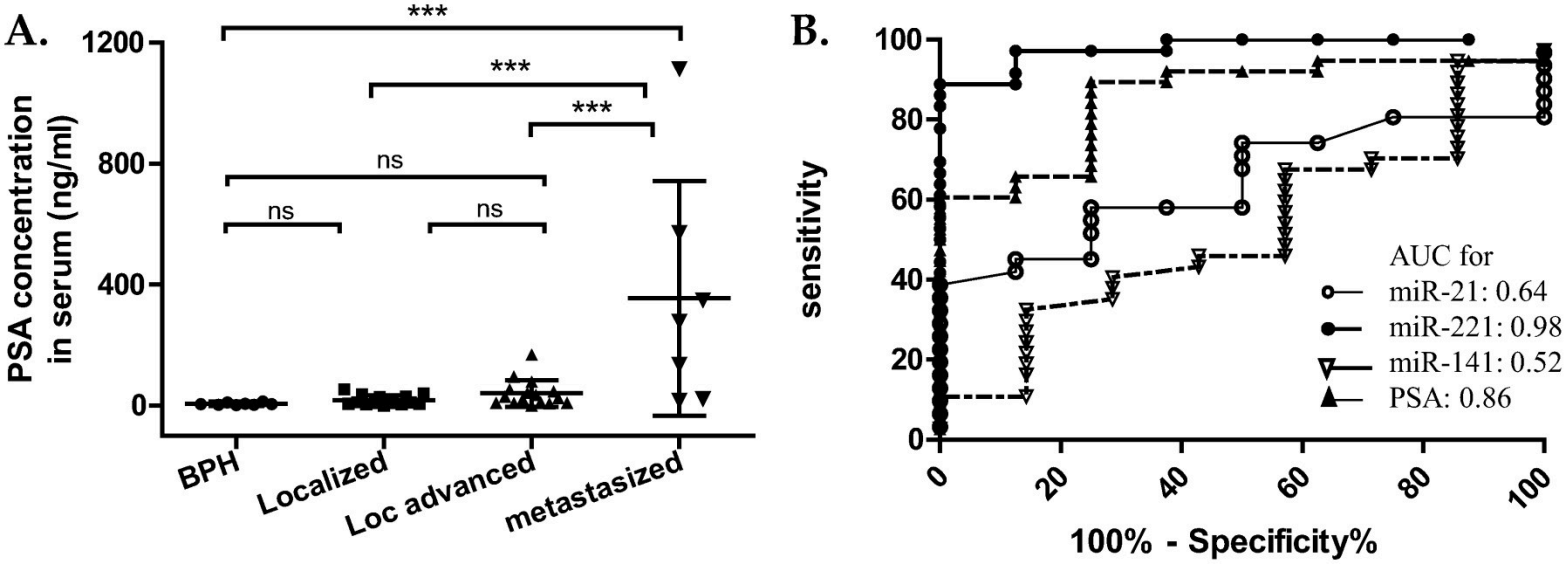
Comparison of PSA with micro-RNA in EVs. (A) PSA level in serum from patients. Preoperative PSA concentration in serum was used for analysis (S1 Table). Patients were the same participants as in the test for plasma EVs; ***: p < 0.001 (One-way ANOVA with Tukey’s post hoc analysis). (B) ROC curve of PSA and each micro-RNA for distinguishing BPH from PCa.

Prostate specific antigen (PSA) level was quantified in serum of the patients whose EVs in plasma were analyzed (Fig 5A) [17]. Only the group with metastasized PCa showed significant difference in the level of PSA from the control group and other PCa groups (P<0.001). There was no significant difference between the control group and local or local advanced group, which means the level of PSA is not discriminative in the state of less progressed disease but distinct in the far progressed state.

In the receiver operating characteristic curve (ROC) about the predictability for prostate cancer, the area under the curve (AUC) for miR-221 in EVs from plasma was 0.98 (95% CI: 0.94 to 1.02, p < 0.0001), while AUC for PSA was 0.86 (95% CI: 0.73 to 0.98, p < 0.01) (Fig 5B). This suggests that miR-221 in EVs from plasma separated the PCa group from the BPH group better than PSA concentration did in serum. The AUC for miR-21 and miR-141 was 0.64 and 0.52, respectively and lower than that of PSA.

## Discussion

Ultracentrifugation is a conventional method for isolating EVs, but it is not appropriate for clinical application that deals with many samples simultaneously. In this study, we evaluated three methods of EV isolation before dealing with clinical samples and used the most appropriate method in the subsequent analysis. This method was compared with other methods both quantitatively and qualitatively, and the EVs isolated by it were characterized.

In the clinical environment, isolating EVs needs additional work after drawing blood from patients. However, since they are released from almost every cell with the information of their original cells, isolating and analyzing their contents could have the effect of enriching useful information. Being based on this, we isolated EVs from patients’ samples, using a clinically practical method.

In the diagnosis of prostate cancer, PSA is a well-known molecule that signifies the possibility of the disease [17]. However, the level of PSA does not give enough information for the disease, and there have been needs for additional molecules that would help liquid biopsy. Many studies handled micro-RNA for diagnostic application, but we focused on micro-RNA in EVs.

Among the three micro-RNAs that we selected, miR-21 is known to function in cell proliferation and relate to cancers [18, 33, 34]; miR-221 is known to regulate the cell cycle [18, 19, 35]; miR-141 is known to affect the expression of androgen receptor [7, 34] and progress of metastatic cancer. In this study, miR-221-3p in localized PCa group showed significantly higher expression than the BPH group, and AUC for miR-221-3p was higher than AUC for PSA. Since it is critical to detect prostate cancer in the early stage where PSA is not discriminative between patients, the information from miR-221 could be applied usefully in this perspective.

## Conclusions

Isolating EVs from plasma had the benefit of concentrating micro-RNAs. Among miR-21, miR-141, and miR-221, miR-221 in EVs from plasma of localized PCa group showed a significantly different expression from the control group than what PSA level in serum had in the same groups. The ability of miR-221-3p discriminating prostate cancer from benign prostatic hyperplasia was better than PSA level in serum. This study suggests that the use of EVs can help develop diagnostic application of micro-RNA, when combined with an appropriate EV isolation method.

## Supporting information

S1 Table Characteristics of patients for analysis of plasma EVs. (DOCX)

S1 Raw images (PDF)
