# Supplementary material for: Evaluation of micro-RNA in extracellular vesicles from blood of patients with prostate cancer
Source: PLoS One. 2021 Dec 31;16(12):e0262017. doi: 10.1371/journal.pone.0262017 (PMC8719659; doi:10.1371/journal.pone.0262017)
Supplement: S1 Table — (DOCX) [file pone.0262017.s001.docx]

**Table S1. Characteristics of patients for analysis of plasma EVs**

|  | BPH | Localized PCa | Local Advanced PCa | Metastsized PCa |
| --- | --- | --- | --- | --- |
| Number of patients | 8 | 15 | 16 | 7 |
| Preoperative PSA level in serum(ng/ml) | 5.51±4.03 | 17.38±15.45 | 39.86±42.2 | 354.22±359.03 |
| Gleason score | - | 7.13±0.62 | 7.94±1.03 | 9±0.53 |
| Mean age(years) | 79.13±1.83 | 70.93±6.58 | 70.13±4.14 | 71.43±3.46 |
| Mean BMI(Kg/m^2^) | 22.04±1.83 | 23.70±2.83 | 24.24±3.88 | 23.12±2.62 |
