# Supplementary material for: Evaluation of micro-RNA in extracellular vesicles from blood of patients with prostate cancer
Source: PLoS One. 2021 Dec 31;16(12):e0262017. doi: 10.1371/journal.pone.0262017 (PMC8719659; doi:10.1371/journal.pone.0262017)
Supplement: S1 Raw images — (PDF) [file pone.0262017.s002.pdf]

Image was inverted in the figure 2.

Image was scanned by Li-CoR Biosciences C-digit Blot scanner.

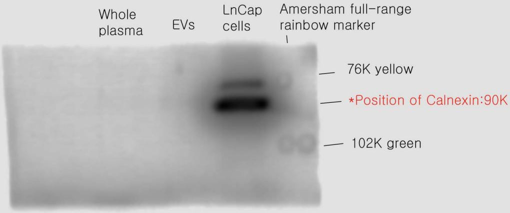

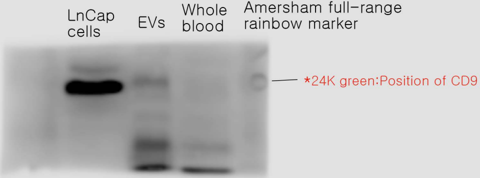

Image scanned by Li-CoR biosciences C-Digit scanner
This image was used in figure 2.

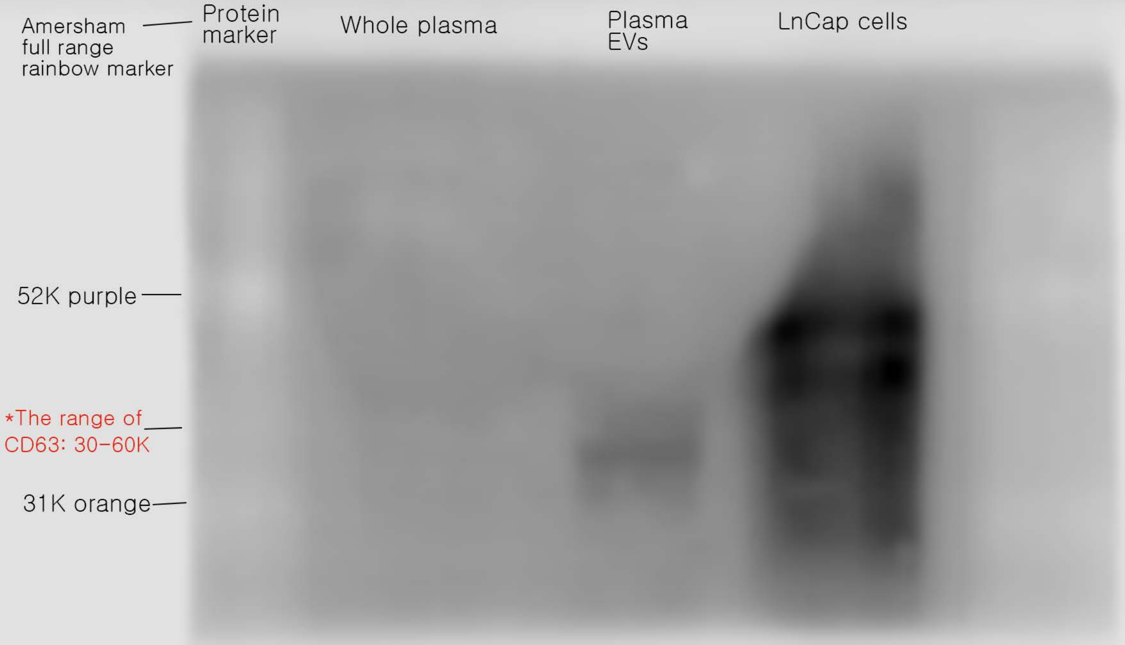

Images scanned by Li-CoR Biociences C-Digit Blot scanner

This image was used in figure 2.

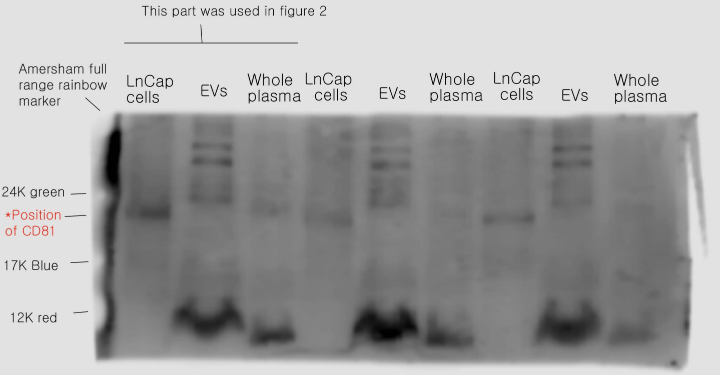

Image scanned by Li-CoR Biosciences C-digit Blot scanner

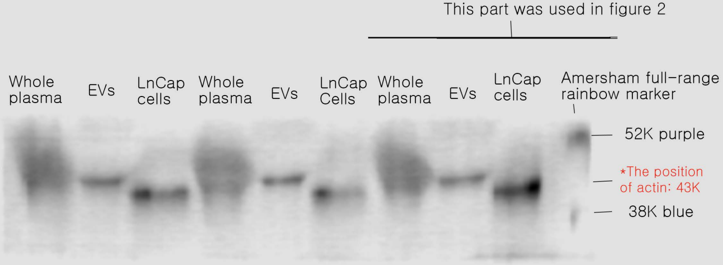

Image scanned by Li-CoR Biosciences C-digit Blot scanner
